# Factors associated with non-optimal antiretroviral adherence among MSM and women living with HIV in South India: an exploratory analysis

**DOI:** 10.1017/S0950268825100927

**Published:** 2026-01-02

**Authors:** Casey Morgan Luc, Sabitha Gandham, Sierra Upton, Vijay Yeldandi, Kara Herrera, Mark S. Dworkin

**Affiliations:** 1 University of Illinois Chicago School of Public Health, USA; 2 SHARE India, India

**Keywords:** antiretroviral adherence, global health, India, MSM, sex work, women living with HIV

## Abstract

Hyderabad, the fourth-most populous city in India, accounts for the majority of people living with human immunodeficiency virus (HIV) (PLWH) in Telangana, likely comprised of two populations with a disproportionately high national HIV prevalence: gay, bisexual, and other men who have sex with men (MSM) and those who engage in sex work (SW). Research has shown that engaging in SW increases vulnerability to HIV transmission risk for both women and MSM, but less is known about contributors to non-optimal (ART) adherence. We analyzed data from 45 MSM and 49 women living with HIV who were enrolled in the first year of data collection from an mHealth education study in Hyderabad. Modified Poisson regression was used to measure factors associated with ART adherence measured with a visual analogue scale (VAS) (model 1) and pill count (model 2). Less than half (40.9%) reported ever engaging in SW, including 13 women and 25 MSM. The prevalence of non-optimal ART adherence was 14.9% with VAS and 42.4% with pill count. Engaging in SW was not associated with non-optimal ART adherence. Differences in non-optimal ART adherence measured by VAS and pill count suggest that future studies should utilize both methods to better distinguish the measures.

## Introduction

The HIV epidemic in India disproportionately impacts gay, bisexual, and other men who have sex with men (MSM) and those who engage in sex work (SW). Among MSM, the nationwide human immunodeficiency virus (HIV) prevalence of an estimated 2.7% is higher than the general population in India [1]. Among MSM living with HIV in India, fewer who know their HIV status are on antiretroviral treatment (ART) compared to the general population [6]. Similarly, the HIV epidemic in India disproportionately impacts SWs with existing surveillance suggesting a higher HIV prevalence than the general population in India [1]. Most SWs in India are female, but engagement in SW among MSM is high [2].

Telangana, a state in the south-central region of India, accounts for more than twice the nationwide adult prevalence of HIV (Telangana: 0.7%; India overall: 0.2%) [3]. Hyderabad, the fourth-most populous city in India, accounts for the majority of PLWH in Telangana with a likely high proportion of MSM and SWs [3–6]. A large subgroup of women living with HIV is likely engaging in SW (National AIDS Control Organization 2021 estimates: 868,000 females who engage in sex work [FSW]), but the proportion of MSM who engage in sex work (MSW) is unknown. Engaging in SW has been shown to increase vulnerability to HIV transmission risk for both women and MSM [7–10], as HIV rates are disproportionately higher among MSW and FSW than MSM and women respectively [9–13].

National estimate for viral suppression among MSM on ART is unknown, with published studies in India within a metropolitan study setting reporting viral suppression levels ranging from 10% to 61% [14–16]. Among women living with HIV, data derived from a systematic review suggests prevalence of ART adherence is higher than men (87% vs. 68%) [1]. Factors such as low HIV health literacy and/or low education may partially contribute to non-optimal ART adherence among women and MSM living with HIV [15, 17–19].

Less is known about ART adherence among MSWs and FSWs living with HIV. MSWs are an important, yet understudied population that may be susceptible to non-adherence to ART due to challenges in healthcare access [2, 8], but no research has measured ART adherence among MSWs living with HIV. One study among FSWs living with HIV in Hyderabad, India, measured ART adherence to be 52% [9], which is likely lower than ART adherence among all women living with HIV [1]. This research measured factors associated with ART adherence among two stigmatized and hard-to-reach populations, MSM and women living with HIV. Since low health literacy may contribute to non-optimal ART adherence [15, 17–19], this research was conducted among those specifically with low education and/or low health literacy.

## Methods

### Sample and recruitment

We analysed baseline data from an mHealth education study to assess the acceptability and feasibility of a mobile phone application among PLWH and low literacy. The application was previously designed in the United States to promote ART adherence and retention in care using a realistic talking Avatar among PLWH. This exploratory study aims to make this application generalizable to populations outside the United States.

In this study, a population of MSM and women living with HIV were recruited. Eligibility criteria included ≥ 18 years of age, English- or Telugu-speaking, diagnosed with HIV, prescribed ART medication for at least 6 months prior to enrolment, and completed an education at a class 10 level in India or lower, or received a score of 12 or lower (level of inadequate or problematic) on the European Health Literacy Questionnaire (HLS-EU-Q16) [20]. For MSM living with HIV, participants reported having ever had at least one male sexual partner. Engaging in SW was determined as responded ‘Yes’ to: ‘Have you ever made any money from sex work’?

Participants were recruited in-person in Hyderabad, India, at one of three non-governmental organizations (NGO) partnered with SHARE India. These NGOs included: 1) DARPAN, serving exclusively MSM, 2) ROSE, serving exclusively FSW, and 3) HOPES, serving women living with HIV (FSW and non-FSW). A member from SHARE India travelled to each of the three NGOs on a rotating schedule during the recruitment period. As NGOs were aware of the SHARE India researcher’s visiting schedule, members from the NGO-served community were present on site for recruitment. At the study site, a trained researcher fluent in both Telugu and English administered the screening questionnaire to determine eligibility and conducted the interviews. Each interview was held in a private space at the NGO facility and lasted 60–90 min. Using a laptop or smartphone, the researcher asked interview questions to participants and recorded their responses in Qualtrics in real time (sample question: ‘How confident are you in taking your medication as prescribed when unexpected events occur’?). In addition, interviews were audio-recorded on a secure device and stored on the researcher’s laptop for review as needed.

Between July 2023 and July 2024, 119 participants were screened, of whom 98 were deemed eligible. A convenience sample of 94 MSM and women living with HIV completed interviews using the structured questionnaire (four eligible participants declined). Participants were compensated 700 Indian rupees (equivalent to USD $12.20 in July 2023) for completing the interview.

### Measures

The structured questionnaire collected the following measures:

Baseline characteristics include age, study population (MSM, women living with HIV), marital status, number of children, religion, employment status, travel distance to their ART clinic, health literacy, income status, and one question to assess the usefulness of a mobile phone application to supplement their HIV care. Health literacy was assessed with the complete HLS-EU-Q16 (α = .66).

Knowledge of HIV was assessed with four questions (e.g. ‘Do you know the names of your HIV medications’?) (α = .63). Treatment self-efficacy was assessed with two questions from the HIV Adherence Self-Efficacy Scale (HIV-ASES). Reasons for missing medications were asked, including fasting for religious reasons, such as prior/during Puja for Hindus or Ramadan for Muslims, which may affect ART adherence due to the high prevalence of Hinduism and Islam practiced in India [21].

Antiretroviral therapy adherence was assessed with the 7-day visual analogue scale (VAS) [22], a reported easy-to-use tool in low-income countries [22–24]. Secondly, a 30-day pill count was calculated. In India, those receiving their medication from public ART Centres all receive a 30-day pill supply. Participants were asked to bring their ART medication to the interview. During the interview, a trained researcher counted the number of available pills in their medication bottle and asked clarifying questions to determine their 30-day adherence (e.g. ‘when did you start taking this medication’?). Viral suppression was collected from viral load data found in the participant’s Green Book. The Green Book is an identity document issued in India to patients seeking care, such as PLWH visiting their clinic, that details patient information, including historical viral load data. The most recent viral load measurement in the Green Book was recorded.

### Ethical considerations

Written informed consent was received from participants. The India Institutional Review Board and the University of Illinois Chicago Institutional Review Board both approved the research under protocol #2021–1,128, including the compensation to participants.

### Analysis

Descriptive statistics were used to summarize characteristics of the study sample. Health literacy was scored as inadequate, with a total score of eight or below, or problematic/sufficient with a score of at least nine, for those eligible with reported low education [20]. A monthly income level of 9,000 rupees or less (equivalent to $3.65 per day, the World Bank’s median poverty line) was defined as the poverty level [25]. For treatment self-efficacy, scores ranged from 0 (cannot do at all) to 10 (completely certain can do) and were averaged between the two questions. Results of the VAS and 30-day adherence percentages were dichotomized as optimally adherent (responding 10, or 100% adherence) and or non-optimal adherence (9 or less, or less than 100%) [23, 26–28].

A modified Poisson regression analysis was conducted with a sandwich estimator of variance to obtain prevalence ratios (PRs) with their 95% confidence intervals (95% CI). Modified Poisson regression is a reliable approach for prospective studies with binary outcome data and has documented use in cross-sectional studies assessing among PLWH [29]. Separate multivariable models were constructed to examine associations between factors and two outcomes: self-reported ART adherence (model 1) and ART adherence assessed through pill count (model 2). The backward selection process was used. This method fits multivariable models with predictive study variables through a process of backwards elimination to assess study variable inclusion (α = .05). This process was used to assess for study variable inclusion due to the limited literature of known factors associated with non-optimal ART adherence among these two understudied populations. Engaging in SW was adjusted a priori. A complete case analysis approach was used. Due to the low number of those with a viral load collected within 6 months prior to interview completion (N = 49), viral suppression was described but not modelled as an outcome. The analyses were performed using Stata software (v. 18).

## Results

### Sample characteristics

Sample characteristics of the participants are presented in Table 1. Among the 94 PLWH in our sample, there were 45 MSM (47.9%) and 49 women (52.1%). A total of 38 (40.9%) reported ever engaging in SW, including 13 women (34.2%) and 25 MSM (65.8%). About half of the participants were never married/separated/widowed (51.1%), had at least one child (55.4%), most had an income above the poverty level (76.6%), were employed or a student (68.1%), and practiced Hinduism (79.7%). Less than half reported an inadequate health literacy score (40.6%); the average antiretroviral treatment knowledge score was 2.7 out of 4, and the average treatment self-efficacy (two questions from HIV-ASES) was 9.6 out of 10. Regarding the mobile phone application, four-fifths (81.9%) of the sample responded with the highest level of usefulness (very useful, 10 out of 10) for a mobile phone application that would function to supplement their HIV care. Nine out of 10 participants (88.4%) commuted to their ART Centre in over 30 min. Time to ART centre was significantly shorter among those engaging in SW compared to those who were not (*P* < .05, Table 2).

**Table 1.** Characteristics of men who have sex with men and women living with human immunodeficiency virus in South India

Study variable^a^	N (%)
ART adherence (visual analogue scale)	
100% (10 out of 10)	80 (85.1)
<100% (9 or less)	14 (14.9)
ART adherence (30-day pill count)	
>90%	49 (57.7)
≤90%	36 (42.4)
Viral suppression	
Target not detected (TND)	79 (90.8)
Detectable	8 (9.2)
Study population	
MSM	45 (47.9)
Women	49 (52.1)
Engaged in sex work	38 (40.9)
Health literacy	
Inadequate	37 (39.5)
Problematic/sufficient	57 (60.5)
Age (Years)	36.2 ± 9.5
Marital status	
Married/living together	46 (48.9)
Never married/separated/widowed	48 (51.1)
Number of children	
0	42 (44.7)
1	23 (24.5)
2+	29 (30.9)
Antiretroviral treatment knowledge (0–4)	2.7 ± 1.3
Treatment self efficacy (HIV-ASES Score, 0–10)	9.6 ± 1.2
Income	
Above poverty level	72 (76.6)
At or below poverty level	22 (23.4)
Employment status	
Employed/student	64 (68.1)
Unemployed/unable to work	30 (31.9)
Travel time to ART Centre	
Less than 30 minutes	10 (10.6)
Between 30 minutes and 1 hour	54 (57.5)
More than 1 hour	29 (30.9)

a Column N may not sum to Total N due to Missingness.

**Table 2.** Characteristics of men who have sex with men and women living with human immunodeficiency virus in South India stratified by study population

Study variable^a^	MSM, N = 45	Women, N = 49	*P*
Non-optimal ART adherence (<100%, VAS)	6 (13.3)	8 (16.3)	.684
Non-optimal ART adherence (≤90%, Pill Count)	17 (41.5)	19 (43.2)	.873
Engage in sex work	25 (55.6)	13 (27.1)	.005
Health literacy			.469
Inadequate	16 (35.6)	21 (42.9)	
Problematic/sufficient	29 (64.4)	28 (57.1)	
Age	35.4 ± 1.6	36.9 ± 1.12	.453
Married/living together	16 (35.6)	30 (61.2)	.013
Antiretroviral treatment knowledge	2.9 ± 0.2	2.7 ± 0.2	.389
Treatment self-efficacy	9.5 ± 0.2	9.7 ± 0.1	.440
Income			.795
Above federal poverty level	35 (77.8)	37 (75.5)	
At or below federal poverty level	10 (22.2)	12 (24.5)	
Employment status			<.001
Employed/student	40 (88.9)	24 (48.9)	
Unemployed/unable to work	5 (11.1)	25 (51.0)	
Number of children			<.001
0	31 (68.9)	11 (22.5)	
1	4 (8.9)	19 (38.8)	
2+	10 (22.2)	19 (38.8)	
Distance to ART Centre			.023
Less than 30 minutes	8 (18.2)	2 (4.1)	
Between 30 minutes and 1 hour	27 (61.4)	27 (55.1)	
More than 1 hour	9 (20.5)	20 (40.8)	

a Column N may not sum to Total N due to Missingness.

The prevalence of non-optimal ART adherence using VAS was 14.9% (Table 1). Of the 94 PLWH who completed the interview, 85 participants (90.4%) were eligible to complete the pill count, and 87 participants (92.6%) had any viral load data from their Green Book to be reported. The prevalence of non-optimal ART adherence using pill count was 42.4% (Table 1).

**Figure 1.**
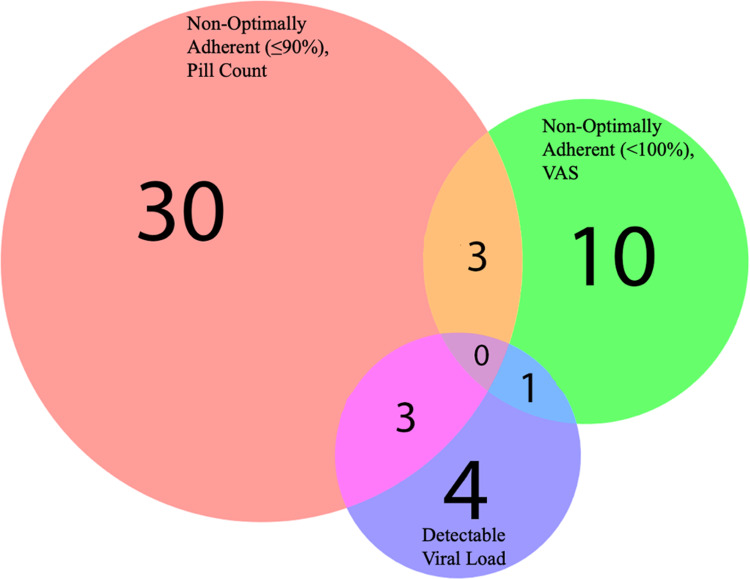
Venn diagram of study outcomes among those who are non-optimally adherent and/or have a detectable viral load.

About half of the participants had a viral load collected within 6 months (55.2%), 28.7% between 6 months and 1 year, 12.6% between one and 2 years, and 3.5% greater than 2 years. Eight participants (9.2%) had a detectable viral load, with four of these viral loads collected within 6 months. One participant who self-reported non-optimal ART adherence (VAS) had a viral load that was detectable, and three participants with a non-optimal ART adherence measured with pill count had a viral load that was detectable (Figure 1). Only three participants reported non-optimal ART adherence, both with VAS and pill count. No participant was both non-optimally adherent to ART using both measures with a detectable viral load.

Our recruitment yielded a significant difference in engaging in SW between MSM and women (*P* < .01), with other notable lifestyle differences between these groups, including marital status, employment status, number of children, and distance to the ART Centre (Table 2).

**Figure 2.**
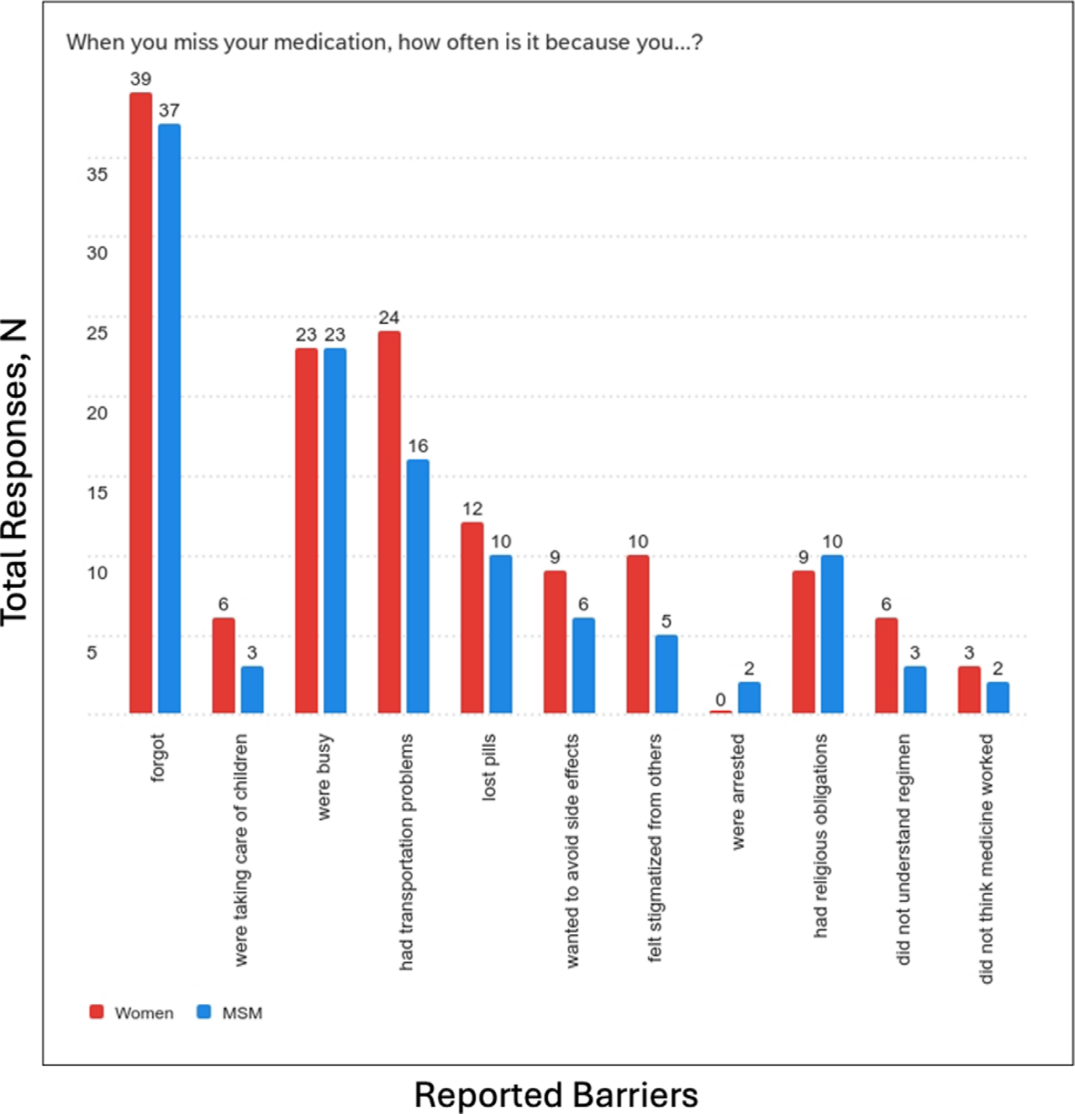
The Most Commonly Reported Reasons for Missing Medication Among Sample Population.

Non-optimal ART adherence was similar comparing MSM and women for both VAS (*P =* .684) and pill count (*P =* .873) (Table 2), as well as between those who engage in SW and do not (VAS, *P =* .671; pill count, *P =* .655, Table 3). Most participants (76.0%) reported at least two reasons for missing their medications. The most commonly reported reasons for missing medication were forgetting to take medication (80.9%), followed by being busy (48.9%) and having transportation problems (42.6%). Reported reasons for missing medication were similar between women and MSM (Figure 2).

**Table 3.** Characteristics of men who have sex with men and women living with human immunodeficiency virus in South India stratified by sex work status

Study variable^a^	Engaged in sex work, N = 38	No sex work, N = 55	*P*
**Outcomes**			
Non-optimal ART adherence (<100%, VAS)	5 (13.2)	9 (16.4)	.671
Non-optimal ART adherence (≤90%, pill count)	16 (45.7)	19 (54.3)	.655
Health literacy			.154
Inadequate	18 (47.4)	18 (32.7)	
Problematic/sufficient	20 (52.6)	37 (67.3)	
Age	35.2 ± 1.4	36.9 ± 1.4	.417
Married/living together	17 (44.7)	28 (50.9)	.558
Antiretroviral treatment knowledge	2.8 ± 0.2	2.8 ± 0.2	.912
Treatment self-efficacy	9.5 ± 0.3	9.6 ± 0.1	.616
Income			.323
Above federal poverty level	31 (81.6)	40 (72.7)	
At or below federal poverty level	7 (18.4)	15 (27.3)	
Employment status			.080
Employed/student	30 (78.9)	34 (61.8)	
Unemployed/unable to work	8 (21.1)	21 (38.2)	
Number of children			.617
0	19 (50.0)	22 (40.0)	
1	8 (21.1)	15 (27.3)	
2+	11 (29.0)	18 (32.7)	
Distance to ART Centre			.010
Less than 30 minutes	7 (18.4)	3 (5.6)	
Between 30 minutes and 1 hour	25 (65.8)	28 (51.9)	
More than 1 hour	6 (15.8)	23 (42.6)	

a Column N may not sum to Total N due to Missingness.

### Associations between variables and non-optimal antiretroviral adherence

Unadjusted associations between factors and non-optimal ART adherence are presented in Table 4 (VAS) and Table 5 (Pill Count). In Table 4, prevalence of non-optimal ART adherence was higher among those who were married/living together (PR: 1.88, 95% CI: 0.68, 5.21), employed (PR: 2.81, 95% CI: 0.67, 11.88), had at least one child (PR: 3.04, 95% CI: 0.79, 11.68), had inadequate health literacy (PR: 2.05, 95% CI: 0.77, 5.47), and reported a shorter commute to the ART Centre (PR: 2.26, 95% CI: 0.75, 6.81) but these associations were not statistically significant. For reported reasons for missing medication, the prevalence of non-optimal ART adherence was highest among those who reported feeling stigmatized by others (PR: 1.44, 95% CHI: 0.45, 4.57) and religious obligations (PR: 1.58, 95% CHI: 0.55, 4.51), But these were not statistically significant. Among associations with pill count in Table 5, prevalence of non-optimal ART adherence was higher among those with a shorter commute to the ART Centre (PR: 4.20, 95% CI: 0.64, 27.38), but this association was not statistically significant.

**Table 4.** Characteristics of men who have sex with men and women living with human immunodeficiency virus in South India by self-reported non-optimal antiretroviral adherence (<100%, visual analogue scale)

Study variable	N	(%) with non-optimal ART adherence	Prevalence ratio (95% CI)
Study population			
MSM	45	6 (13.3)	0.82 (0.31, 2.18)
Women	49	8 (16.3)	referent
Engage in sex work	38	5 (13.2)	0.80 (0.29, 2.22)
Health literacy			
Inadequate	37	8 (21.6)	2.05 (0.77, 5.47)
Problematic/sufficient	57	6 (10.5)	referent
Age	–	37.7 ± 2.3	1.02 (0.97, 1.06)
Married/living together	46	9 (19.6)	1.88 (0.68, 5.21)
Antiretroviral treatment knowledge	–	2.4 ± 0.3	0.79 (0.56, 1.12)
Treatment self-efficacy	–	9.5 ± 0.2	0.96 (0.74, 1.24)
Income			
Above federal poverty level	72	11 (15.3)	Referent
At or below federal poverty level	22	3 (13.6)	0.89 (0.27, 2.93)
Employment status			
Employed/student	64	12 (18.8)	2.81 (0.67, 11.88)
Unemployed/unable to work	30	2 (6.7)	Referent
Number of children			
0	42	3 (7.1)	Referent
1+	52	11 (21.2)	3.04 (0.79, 11.68)
Distance to ART Centre			
Less than 30 minutes	10	3 (30.0)	referent
More than 30 minutes	83	11 (13.3)	2.26 (0.75, 6.81)
**Reasons for missing medication**			
Forgot	76	14 (18.4)	undefined
Were Busy	46	8 (17.4)	1.39 (0.52, 3.72)
Transportation problems	40	8 (20.0)	1.80 (0.67, 4.80)
Avoid side effects	15	2 (13.3)	0.88 (0.22, 3.56)
Felt stigmatized from others	15	3 (20.0)	1.44 (0.45, 4.57)
Religious obligations	19	4 (21.1)	1.58 (0.55, 4.51)

**Table 5.** Characteristics of men who have sex with men and women living with human immunodeficiency virus in South India by non-optimal antiretroviral adherence (≤ 90%, pill count)

Study variable	N	(%) with non-optimal ART adherence	Prevalence ratio (95% CI)
Study population			
MSM	41	17 (41.5)	0.96 (0.58, 1.58)
Women	44	19 (43.2)	Referent
Engage in sex work	36	16 (44.4)	1.12 (0.68, 1.87)
Health literacy			
Inadequate	34	14 (41.2)	0.95 (0.57, 1.60)
Problematic/sufficient	51	22 (43.1)	Referent
Age	–	36.7 ± 1.5	1.00 (0.98, 1.03)
Married/living together	41	18 (43.9)	1.07 (0.65, 1.77)
Antiretroviral treatment knowledge	–	2.7 ± 0.2	0.91 (0.75, 1.12)
Treatment self-efficacy	–	9.8 ± 0.1	1.28 (0.93, 1.75)
Income			
Above federal poverty level	68	29 (42.7)	Referent
At or below federal poverty level	17	7 (41.2)	0.97 (0.51, 1.82)
Employment status			
Employed/student	56	20 (35.6)	0.65 (0.40, 1.05)
Unemployed/unable to work	29	16 (55.2)	Referent
Number of children			
0	39	16 (41.0)	Referent
1+	46	20 (43.5)	1.06 (0.64, 1.75)
Distance to ART Centre			
Less than 30 minutes	9	1 (11.1)	Referent
More than 30 minutes	75	35 (46.7)	4.20 (0.64, 27.38)
**Reasons for missing medication**			
Forgot	68	28 (41.2)	0.88 (0.49, 1.57)
Were busy	39	17 (43.6)	1.06 (0.64, 1.74)
Transportation problems	36	14 (38.9)	0.87 (0.52, 1.45)
Avoid side effects	11	4 (36.4)	0.84 (0.37, 1.93)
Felt stigmatized from others	14	6 (42.9)	1.01 (0.52, 1.98)
Religious obligations	18	5 (27.8)	0.60 (0.27, 1.33)

The final sample to assess the adjusted associations was 93 participants for VAS. For the pill count, no adjusted table is presented because all associations between study variables and non-optimal ART adherence were null. Adjusting for determinants, engaging in SW was not associated with non-optimal ART adherence using VAS (Table 6: *P =* .117), similar to the crude association using pill count. In Table 6, low health literacy, having children, being employed, and a shorter commute to the ART centre were significantly associated with non-optimal ART adherence (VAS). (*P* < .05). Additionally, the prevalence of non-optimal ART adherence was 2.8 times higher among the 19 (20.4%) participants who reported missing their ART medication due to religious obligations than those who did not (aPR: 2.88, 95% CI: 1.02, 8.18).

**Table 6.** Multivariable associations of self-reported non-optimal antiretroviral adherence (visual analogue scale, < 100%) among men who have sex with men and women living with human immunodeficiency virus in South India

Study variable^a^	aPR (95% CI)	*P*
Sex work	0.47 (0.18, 1.21)	.117
Health literacy		
Inadequate	3.40 (1.25, 9.29)	.017
Problematic/sufficient	Referent	
Time to ART Centre		
Less than 30 minutes	4.51 (1.16, 17.50)	.030
More than 30 minutes	Referent	
Number of children		
0	Referent	
1+	3.70 (1.30, 10.48)	.014
Employment status		
Employed/student	3.58 (1.03, 12.44)	.035
Unemployed/unable to work	Referent	
**Reasons for missing medication**		
Religious obligations	2.88 (1.02, 8.18)	.047

a Adjusted variables based on backward selection (α = .05); sex work adjusted a priori.

## Discussion

To the best of our knowledge, this is the first study to explore factors associated with non-optimal ART adherence in a convenience sample of MSM and women living with HIV in South India, with a high proportion of MSWs and FSWs in the sample.

The main finding from this exploratory analysis is that non-optimal ART adherence was low-moderate, ranging from 15% using the self-report VAS measure to 42% using pill count. There were no notable differences in adherence between women and MSM. Overall, the magnitude of non-optimal ART adherence was different between the two measures. The VAS self-report may suggest non-optimal ART adherence is low in this exploratory study, but our pill count measure may suggest self-report underestimates non-optimal ART adherence in our study sample, consistent with the literature, as self-report may be subject to social desirability, among other factors [30, 31]. Additionally, we only used one question from the 3-item validated VAS, lowering specificity and possibly resulting in a lower reported non-optimal ART adherence. While VAS has been cited as useful due to its simplicity [24], pill count may more accurately capture ART adherence even though there may be inaccuracies in self-report, such as pills transferred to a participant’s current bottle, possible medication diversion, and/or ‘pill dumping’ to overestimate ART adherence [32].

Additionally, findings from the Venn diagram reveal there is little overlap between those found to be non-optimally adherent to ART by VAS and pill count, as these two groups were fairly distinct. Due to the lack of overlap in these measures in our study sample, it is difficult to recommend a single ART adherence measure after 1 year of data collection. Future work should continue to report both measures to further understand the relative accuracy of these measures. Other strategies to increase the number of well-timed viral loads at or around the interview date may best discriminate between both self-reported adherence measures.

In a review of the literature, only one other study measured ART adherence among MSM in India. Piña et al. found in its combined sample of 65 MSM and transgender persons in Mumbai that 52% were non-optimally adherent to ART after 3 months [17]. For women living with HIV, there are few studies aimed to engage women in HIV care in South India [33–35], with one study by Ekstrand et al. found that in a sample of 600 rural woman living with HIV in South India, only 30% of total ART treatment was taken in the past month. Both studies had a higher overall proportion of self-reported non-optimal adherence than self-reported adherence (VAS) in our study, but rates were similar to pill count adherence, underlining the challenges of adhering to ART regimens among these understudied populations living with HIV in South India.

Among factors, being employed and having children were associated with non-optimal ART adherence using VAS. These factors may be an indicator for demanding schedules that may interrupt daily medication-taking practices. Consistent with this finding, many participants in the study reporting ‘being busy’ as a barrier to adherence, supported by prior research among PLWH in India [36]. Separately, the observed association between low health literacy and non-optimal adherence highlights the need for accessible educational resources to enhance HIV treatment self-efficacy. Our pilot mobile phone application was designed with these needs in mind, offering simplified HIV-related health information and daily medication reminders that may help mitigate both literacy-related barriers and the challenges posed by demanding schedules.

While two studies found fasting was not associated with non-optimal ART adherence [37, 38], our study found that reporting religious obligations was associated with non-optimal ART adherence using VAS. Participants in the cited literature were primarily Muslim, whereas our sample consisted mostly of Hindus, which may partly explain the observed differences. HIV care providers may consider counselling patients on the importance of maintaining adherence to their ART during periods of religious fasting or holidays. Interestingly, a shorter commute to the ART Centre was associated with non-optimal ART adherence for both VAS and pill count, a contradictory finding that warrants further exploration.

Lastly, engaging in SW was not associated with ART adherence for either ART measure. Published literature supports the role of SW to be a risk factor for HIV transmission among MSM and women in India [9–13], but may not be a risk factor for non-optimal ART adherence. Women were less likely to report engaging in SW compared to MSM. Some participants may have indicated employment based on any involvement in SW, and their income reports may have included earnings from it. However, this is less likely given the overall low number of participants reporting engaging in SW in this sample. Among the 13 women who disclosed involvement in SW, only three were willing to specify the type of work (e.g., street-based, secret, or home-based). As a result, we were unable to analyse differences based on the type of SW. Future research should explore strategies to gain participant trust during data collection, such as through additional cultural sensitivity training for interviewers.

## Limitations

This study did not achieve its proposed sample size of at least 200 participants due to local issues that halted data collection in July 2024 for a long enough duration that study staff were lost from the study. Recruitment relied on a convenience sample of MSM and women living with HIV who were connected to NGOs, which may have biased the sample toward individuals who were more engaged in care, socially connected, and adherent to ART compared to those not linked to NGO services. The cross-sectional analysis was conducted as a supplement to a longitudinal study evaluating the efficacy of a mobile phone application to support HIV care among PLWH with low literacy. Due to these limitations, the sample may not be generalizable for MSM and women living with HIV across Hyderabad.

As this study was exploratory and to keep it feasible, we did not assess specific measures such as SW or the HIV-ASES, beyond the relatively sensitive single item (or two items, in the latter case) that we used. Future research should use a multimodal recruitment approach, including digital strategies, to obtain a larger and more representative sample.

## Conclusions

This exploratory study of MSM and women living with HIV in South India, two understudied populations, found low-to-moderate ART adherence with differences between self-reported and pill count measures, underscoring the need for more accurate adherence assessment. Employment and having children were associated with non-optimal adherence. Mobile phone applications offering simplified HIV-related information and daily medication reminders may help address literacy-related barriers and the challenges posed by demanding schedules.

## Data Availability

The data will be made publicly available upon completion of the study. Information on how to access the data may be obtained by contacting the corresponding author.
